# Health Tract, No. 253

**Published:** 1866-06

**Authors:** 


HEALTH TRACT, No. 253.

### NIGHT AND DISEASE.

Sickness and death generally come in the night; it is then, when the body is in a state of weariness from the labors of the day; when, in addition the heavy night-damp has its depressing influences, and the bright sunshine and the light balmy air of all out doors is not present to invigorate and enliven, it is then that the human organism is most susceptible to adverse influences, and is less capable of resisting and warding off the approaches of disease and decay and death. It is the out doors and the sunshine which so oxygenates the blood, and imparts to it a sparkle and a life as it courses through artery and vein; every step becomes a pleasure, every thought a happiness, and it is delicious even to breathe. Now what is it that gives to the out-door air of a clear sunshiny day all these soul thrilling qualities? it is the greater amount of oxygen with which the sun loads every breath we take, which makes all the difference between the joy of the out-door sunshine and the chamber of midnight; and if we could but breathe this highly oxygenated air all the time, men would, other things being equal, double the time of life and be still young in heart and feeling at the age of a hundred years. But, most unfortunately, and not necessarily either, one clear third of our entire existence is spent in breathing a vitiated air, an atmosphere so full of heaviness and dust and odors of a close confined bedroom, as to exclude the more aerial oxygen, hence so many of us, and so often, wake up in the morning with a feeling of tiredness or unrest, that is absolutely distressing, instead of waking up to mirth and laughter and song; a very large part of domestic bickerings which poisons the peace of families arises from the fact, that the parents having slept in an ill-ventilated apartment, have not been refreshed, their sleep has not rested them; every breath taken into the lungs was so impregnated with grosser impurities that it could not take in the more etherial oxygen, whose office it is to absorb the impurities from the blood and carry them out of the system; hence both brain and body are depressed, the moral nature imbibes the evil influence, and both husband and wife wake up to carp, and complain and scold, dampening the spirits of the children, irritating the servants, making a veritable hell in a household which ought to have been a heaven. The importance then of sleeping in an air as pure as possible, socially, physically and morally, can scarcely be over-rated; hence sleep in the highest, largest, best-lighted rooms in the house with open fireplaces and a little fire burning during winter nights, no standing liquids, and with as little carpeting and furniture as possible, in short an almost empty room, unpapered walls, hung with beautiful pictures, paintings and engravings, calculated to elevate the mind, to purify the moral affections, and to giv a direction to thoughts in the beginning of the day, which shall pervade the whole character and conduct, for good, until the pillow is reached again in the early evening.

